# The Effect of Step Width on Muscle Contributions to Body Mass Center Acceleration During the First Stance of Sprinting

**DOI:** 10.3389/fbioe.2021.636960

**Published:** 2021-07-14

**Authors:** Ruoli Wang, Laura Martín de Azcárate, Paul Sandamas, Anton Arndt, Elena M. Gutierrez-Farewik

**Affiliations:** ^1^KTH MoveAbility Lab, Department of Engineering Mechanics, KTH Royal Institute of Technology, Stockholm, Sweden; ^2^KTH BioMEx Center, Royal Institute of Technology, Stockholm, Sweden; ^3^The Swedish School of Sport and Health Sciences, Stockholm, Sweden; ^4^Department of Clinical Science, Intervention and Technology, Karolinska Institutet, Stockholm, Sweden

**Keywords:** sprint biomechanics, induced acceleration analysis, three-dimensional motion analysis, sprinting performance, competitive sprinters

## Abstract

**Background:**

At the beginning of a sprint, the acceleration of the body center of mass (COM) is driven mostly forward and vertically in order to move from an initial crouched position to a more forward-leaning position. Individual muscle contributions to COM accelerations have not been previously studied in a sprint with induced acceleration analysis, nor have muscle contributions to the mediolateral COM accelerations received much attention. This study aimed to analyze major lower-limb muscle contributions to the body COM in the three global planes during the first step of a sprint start. We also investigated the influence of step width on muscle contributions in both naturally wide sprint starts (natural trials) and in sprint starts in which the step width was restricted (narrow trials).

**Method:**

Motion data from four competitive sprinters (2 male and 2 female) were collected in their natural sprint style and in trials with a restricted step width. An induced acceleration analysis was performed to study the contribution from eight major lower limb muscles (soleus, gastrocnemius, rectus femoris, vasti, gluteus maximus, gluteus medius, biceps femoris, and adductors) to acceleration of the body COM.

**Results:**

In natural trials, soleus was the main contributor to forward (propulsion) and vertical (support) COM acceleration and the three vasti (vastus intermedius, lateralis and medialis) were the main contributors to medial COM acceleration. In the narrow trials, soleus was still the major contributor to COM propulsion, though its contribution was considerably decreased. Likewise, the three vasti were still the main contributors to support and to medial COM acceleration, though their contribution was lower than in the natural trials. Overall, most muscle contributions to COM acceleration in the sagittal plane were reduced. At the joint level, muscles contributed overall more to COM support than to propulsion in the first step of sprinting. In the narrow trials, reduced COM propulsion and particularly support were observed compared to the natural trials.

**Conclusion:**

The natural wide steps provide a preferable body configuration to propel and support the COM in the sprint starts. No advantage in muscular contributions to support or propel the COM was found in narrower step widths.

## Introduction

The best results in sprint running are achieved by developing high forward acceleration, reaching the maximal speed, and keeping that speed over the remainder of the run (Ross et al., 2001). According to Newton’s second law, the acceleration of the body center of mass (COM) can be obtained by dividing the external forces by the mass of the sprinter. The largest external force acting on the system are the forces generated while the foot is in contact with the ground, i.e., the ground reaction force (GRF). Therefore, in order to develop a great amount of forward acceleration, a large forward-directed GRF must be generated. Previous studies report that the largest forward-directed force (Rabita et al., 2015) and the greatest forward acceleration (Nagahara et al., 2019) were produced during the first stance phase of the sprint, i.e., the first step during the acceleration phase. However, it is difficult to intuitively understand and quantify how muscle forces produce joint and body COM accelerations in a 3D multi-joint movement due to the complexity of inter-joint interactions.

Muscles are the major contributors to COM motion and are in charge of accelerating all joints in the body (Zajac and Gordon, 1989). Previous studies have analyzed individual muscle contributions to COM acceleration using mainly two different approaches: an induced acceleration analysis (IAA) and a perturbation analysis. Individual muscle contributions to a joint or COM acceleration in a predefined movement can be computed from the generalized equation of motion. There are two unknowns in the generalized equation: the acceleration induced by any one muscle and the partial GRF induced by this muscle’s force. In IAA (Zajac and Gordon, 1989), the partial GRF is commonly computed as a contact force through a kinematic constraint between the foot and the ground, for instance, a rolling constraint with no translations or vertical twist. Perturbation analysis is a technique that can be used to determine the effect that a small change to one or more parameters has on the solution. By perturbating each muscle force slightly, e.g., by adding 1N, and a forward simulation was performed during a very short period of time, from which the changes in position and acceleration of the COM related to that force contributor in gait were examined (Liu et al., 2006). Compared to perturbation analysis, IAA can identify the instantaneous effect of individual muscle force and has a great advantage in computational efficiency. Hamner et al. (2010) and Hamner and Delp (2013) applied IAA to study muscle contributions to COM acceleration in participants running on a treadmill at a range of speeds. They report that the soleus was the main contributor to forward and vertical COM acceleration, regardless of the speed. Debaere et al. (2015) used perturbation analysis to compute muscle contributions to COM acceleration during the first two stance phases of sprint running, and also found the soleus to be the major contributor to forward and vertical COM acceleration. However, perturbation analysis is sensitive to the stiffness of the foot-ground contact model and, due to the need of very stiff foot springs, a weld constraint was simulated in their study. A rolling constraint has been suggested as more realistic (Hamner et al., 2013). Muscle contributions to COM acceleration using IAA during the first step of sprint running have not yet been studied.

Analyses of muscle contributions to COM acceleration have practically only focused on the sagittal plane. There is, however, motion outside of the sagittal plane; this is particularly evidenced by the variation of the step width over a sprint run (Ito et al., 2006; Nagahara et al., 2017). Even though the effect of step width has been quantitatively studied in only a few studies, Ito et al. (2006) recommended that sprinters start their run with wide steps in order to generate greater propulsive forces, then decrease step width gradually over the next few steps, as running speed increases and contact time with the ground decreases. Nagahara et al. (2017) concluded that a wider step width would generate a greater mediolateral impulse. Sandamas et al. (2019) also found the same trend regarding mediolateral impulse in an experimental study comparing a natural wide step length and a restricted narrower step width during sprint start. Greater anterior toe-off velocity and mediolateral motion of the COM were observed in trials with wider step widths. In trials with narrower step widths, lower medial GRFs were generated, but no differences in normalized average antero-posterior power nor in sprinting performance in terms of anterior toe-off velocity were observed. To the best of the authors’ knowledge, no study has previously used IAA to analyze whether step width can influence sprinting performance. The aim of this study is thus to investigate how individual lower limb muscles contribute to COM acceleration in three global directions during the first sprint step in runners’ natural wide step, and second, to determine how these induced accelerations are influenced when step width was restricted.

## Materials and Methods

### Participants

Four (2 male and 2 female) competitive sprinters (mean ± standard deviation: height, 1.75 ± 0.10 m; mass, 70.25 ± 14.04 kg) participated in the study. The personal best for 100 m for two male sprinters is 10.98 and 11.30 s, and 11.47 and 11.93 s for two female sprinters. The sprinters are a sub-cohort of a previous study (Sandamas et al., 2019). The Stockholm Regional Ethical Committee approved the study and participants provided written consent.

### Procedure

**FIGURE 1:**
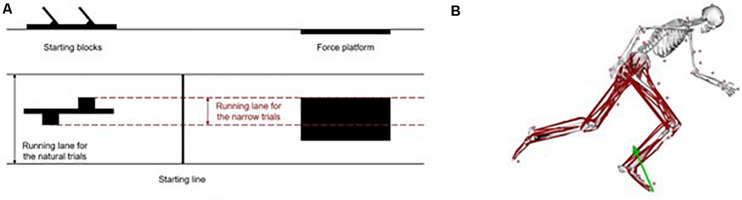
**(A)** Front (top) and top (bottom) views of the setup of the laboratory for the narrow trials. Solid black lines represent the whole running track surface and the dashed red lines represent the limits within the sprinter could run during the narrow trials. **(B)** Musculoskeletal model with 74 markers, 22 body segments and 80 Hill-type musculotendon actuators used during the simulations with OpenSim of the first stance phase of sprint running. The green arrow represents the GRF.

Each athlete performed a total of ten sprint start trials with 74 reflective markers placed on the body. Each sprinter performed five sprint trials in their natural style and other five trials during which they were asked to stay within a 30-cm lane, indicated by ropes on the floor (Figure 1A). For each sprinter, the position of the starting blocks with respect to the force plate was adjusted for natural and narrow trials, respectively. From these 10 trials, two trials per sprinter were chosen for further analysis: the sprint start with the widest (“natural trial”) and the narrowest (“narrow trial”) step width. The running track was 15 m long with a crash mat on the end of the wall. The procedure has been described in greater detail in previous studies (Sandamas et al., 2019, 2020).

Marker trajectories were sampled at 250 Hz with a twelve-camera motion capture system (Oqus 4, Qualisys AB, Gothenburg, Sweden). A force platform (Kistler Group, Winterthur, Switzerland) was embedded in the floor and covered with a running tartan surface. GRF during the stance phase of the first step was recorded at 1500 Hz and a force threshold of 10 N was defined in the vertical direction. Kinematics and kinetics data were low-pass filtered at 50 Hz with a fourth-order Butterworth filter using custom designed scripts (Matlab R2017b, MathWorks Inc., United States). The high cut-off frequency (12 Hz) was used for both data in order to avoid the development of artificial peaks in joint moments due to the loss of the high frequency content in the kinematic data, which was usually filtered at a lower cut-off frequency. Electromyography (EMG) patterns were measured with surface electrodes (Noraxon U.S.A., Inc., Scottsdale, AZ, United States) placed bilaterally over six lower-limb muscles: soleus, gastrocnemius medialis, biceps femoris (caput longum), vastus lateralis, gluteus medius, and gluteus maximus. The raw EMG signals were recorded at 1500 Hz, band-pass filtered between 30 and 300 Hz, rectified, low-pass filtered with a cut-off frequency of 6 Hz, and normalized to the maximal value found during the whole recording (Supplementary Material B). Second-order Butterworth filters were used. Normalization between 0 and 1 was based on the maximum voltage recorded for each muscle and subject.

### Musculoskeletal Simulations

Musculoskeletal simulations were performed [OpenSim v3.3 (Delp et al., 2007)] based on a generic musculoskeletal model with 22 rigid body segments and 37 degrees of freedom that was developed by Rajagopal et al. (2016) (Figure 1B). Segments included a combined head and torso, a pelvis, and a right and left femur, patella, tibia/fibula, talus, calcaneus, toes, humerus, ulna, radius, and hand. Of the model’s 37 degrees of freedom, 20 corresponded to the lower body (six for the pelvis and seven per leg) and were driven by 80 Hill-type musculotendon actuators with the Millard equilibrium muscle model (Millard et al., 2013) and with the enhancements from Lai et al. (2017) to the model for sprinting studies. The remaining 17 degrees of freedom related to the upper body (three for the back and seven per arm) were driven by torque actuators.

The simulation was performed using a dynamic simulation pipeline which consisted of steps for scaling, inverse kinematics, inverse dynamics, residual reduction, computed muscle control and IAA (Hamner et al., 2010). The generic model was first scaled to fit the anthropometry of each participant by using the marker positions collected during the standing reference trial (Delp et al., 2007). Joint angles were generated with an inverse kinematics algorithm (Delp et al., 2007), which minimized the sum of weighted squared position errors between the experimental markers and their corresponding virtual markers. Joint moments were computed with the inverse dynamic algorithm, for which data from the simulated kinematics and the measured GRF were used to solve the equations of motion. Thereafter, the residual reduction algorithm (Delp et al., 2007) was carried out in order to reduce dynamic inconsistencies between kinematics and kinetics due to modeling assumptions and errors when processing motion capture data. The algorithm minimized the sum of weighted squared actuator controls and acceleration errors, using the CFSQP (C functions of sequential quadratic programming) optimizer to solve the optimization problem. Muscle forces, excitations and activations were then calculated with the computed muscle control algorithm (Delp et al., 2007), which solved the muscle redundancy problem by minimizing the sum of squared excitations of muscles and actuators. EMG signals were used to constraint soleus, gastrocnemius medialis, vastus lateralis, biceps femoris, gluteus medialis and gluteus maximus muscles, so that their simulated activations closely matched the recorded activations. Finally, IAA was performed and the contributions of each muscle to the COM acceleration in the three global directions were estimated. The interaction between the foot and the ground was simulated as a rolling constraint and, consequently, the foot could not penetrate the ground, slip in the transverse plane, nor twist along the longitudinal axis. Note that that induced accelerations could not be analyzed during the first 0.03 s and the final 0.04 s – approximately the first 14% and the last 18% of the stance phase – due to limitations in the computed muscle control and induced acceleration analysis; abrupt changes in muscle forces when initializing the muscle states with the computed muscle control tool, and the sudden drop to zero of the induced reaction constraints simulating the foot-floor model due to the limited accuracy of the rolling constraint at the end of the stance phase, made it impossible to converge to a solution during these periods.

### Data Analysis

The cumulative contributions of individual muscles to COM acceleration were calculated by summing the induced acceleration between 14 and 82% of the stance phase. The induced accelerations at the joint level were then computed by summing the cumulative accelerations induced by all muscles spanning that joint. The summed contributions of all muscles spanning the ankle, knee, and hip joint were thus computed.

Step width and COM velocity were calculated with a 15 segment (head, trunk, pelvis, and right and left humerus, ulna/radius, hand, femur, tibia/fibula, and foot) model (Visual3D v6, C-Motion Inc., Germantown, MD, United States). Step width was calculated as the mediolateral distance between the midpoints of markers located at the first and fifth metatarsal head on the right and left feet when the front foot was on the starting blocks and the rear foot contacted the ground for the first time (Otsuka et al., 2014; Sandamas et al., 2019). COM velocity was defined as the time derivative of the whole body COM position based on the COM locations and masses of individual body segments.

## Results

### Measured GRF and the Spatio-Temporal Characteristics

**FIGURE 2:**
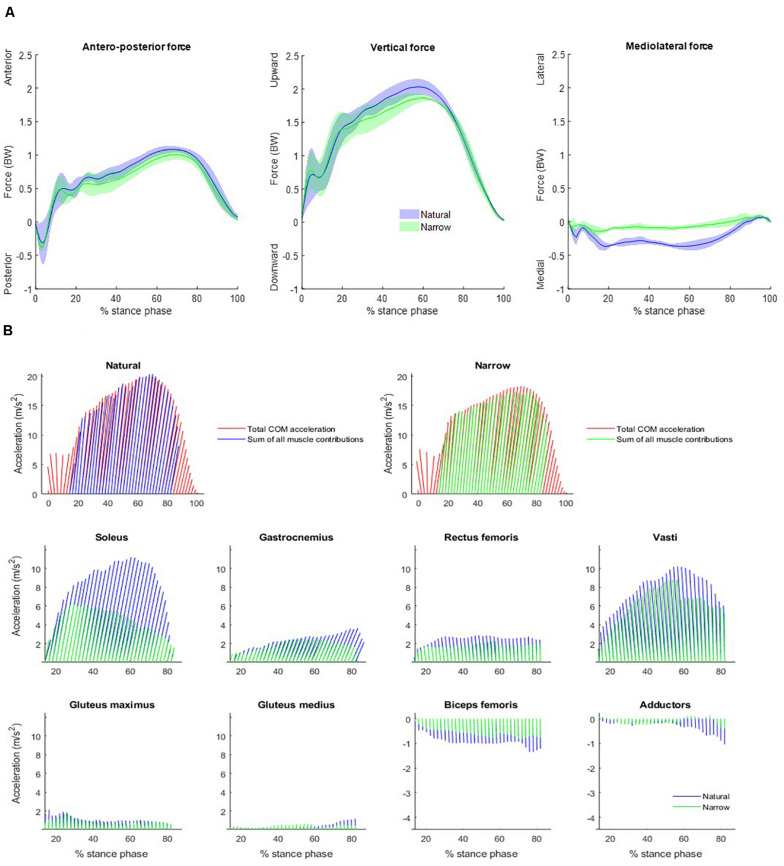
**(A)** Measured GRF averaged across subjects over the stance phase. Each plot includes data of the natural (blue) and the narrow (green) trials. Solid lines represent the mean values and the colored areas represent the mean ± SD values. Forces are measured in body weight (BW). **(B)** Forward and vertical net accelerations and individual muscle contributions to body COM acceleration over the stance phase, averaged over all participants, shown as vectors at every 2% of the stance phase between 14 and 82% of the stance phase. Net accelerations (top) are the average of each participant’s measured GRF divided by his/her mass (red) and the sum of all the muscle contributions across the natural (blue) and the narrow (green) trials. Lower-limb muscles considered were soleus, gastrocnemius (medialis and lateralis combined), rectus femoris, vasti (vastus intermedius, lateralis, and medialis combined), gluteus maximus, gluteus medius, biceps femoris long head, and adductors (adductor brevis, longus and magnus, semitendinosus, semimembranosus and gracilis combined). Note that the scale of the *y*-axis varies among different muscles.

Averaged measured GRF was plotted in the Figure 2A. Peak forward and vertical GRF were lower in the narrow trials than in the natural trials, by approximately 0.20 times body weight (BW) in both directions. Peak rear-directed reaction force occurred at 3% of the stance phase, with the braking phase finishing at approximately 6% of the stance phase, similar in both trials even though the peak force was 0.07 times BW greater (in absolute value) in the narrow trials. GRF was directed more medially in the natural trials then in the narrow trials.

The spatio-temporal characteristics in the natural and narrow trials are summarized in the Table 1A (Supplementary Material A). The mean ± standard deviation step width was 0.37 ± 0.04 m in the natural and 0.15 ± 0.04 m in the narrow trials. The contact phase was an average of 0.02 s longer in the narrow trials than in the natural trials. Normalized average antero-posterior power was smaller in the narrow (0.73 ± 0.08) than in the natural (0.80 ± 0.05) trials.

**TABLE 1:** Comparison of the maximum and mean muscle contributions to forward and vertical COM accelerations in the natural and narrow trials.

	**Maximum contributions (m/s^2^)**						**Mean contributions (m/s^2^)**			
	**Forward**		**Rearward**		**Vertical**		**Forward**		**Vertical**	
	**Natural**	**Narrow**	**Natural**	**Narrow**	**Natural**	**Narrow**	**Natural**	**Narrow**	**Natural**	**Narrow**
Soleus	11.82 (1.91)	8.47 (1.22)	–	–	12.41 (2.41)	8.93 (1.80)	8.06 (1.87)	6.41 (0.81)	8.46 (2.50)	6.36 (0.50)
Gastrocnemius	10.03 (2.40)	8.79 (2.07)	–	–	5.34 (1.15)	4.00 (0.97)	4.70 (2.09)	5.24 (1.08)	2.41 (0.83)	2.45 (0.70)
Rectus femoris	–	–	−1.68 (0.36)	−1.50 (0.88)	3.34 (0.15)	3.72 (0.87)	−1.02 (0.18)	−0.82 (0.34)	2.50 (0.27)	2.82 (0.89)
Vasti	–	–	−4.52 (0.50)	−3.51 (0.63)	10.93 (1.55)	12.68 (2.32)	−2.83 (0.38)	−2.23 (0.37)	7.70 (1.07)	8.67 (0.84)
Gluteus maximus	0.76 (0.81)	0.69 (0.42)	−0.67 (0.84)	−0.23 (0.08)	2.70 (1.06)	3.18 (0.69)	−0.27 (0.32)	0.18 (0.19)	1.12 (0.67)	1.37 (0.41)
Gluteus medius	0.60 (0.32)	0.81 (0.62)	−0.54 (0.39)	−0.35 (0.13)	1.36 (0.39)	2.43 (0.30)	0.01 (0.14)	0.14 (0.26)	0.39 (0.17)	0.55 (0.50)
Biceps femoris	0.70 (0.14)	0.56 (0.18)	−0.11	–	−1.81 (0.22)	−1.68 (0.27)	0.38 (0.10)	0.29 (0.10)	−0.95 (0.09)	−0.85 (0.14)
Adductors	0.42 (0.33)	–	−0.25 (0.12)	−0.73 (0.46)	−1.25 (0.41)	−1.17 (0.26)	0.06 (0.10)	−0.33 (0.26)	−0.32 (0.26)	−0.23 (0.32)

*Averaged and standard deviation (in brackets) values of the contribution of the soleus, gastrocnemius (medialis and lateralis combined), rectus femoris, vasti (vastus intermedius, lateralis and medialis combined), gluteus maximus, gluteus medius, biceps femoris long head, and adductors (adductor brevis, longus and magnus, semitendinosus, semimembranosus, and gracilis combined) for all participants are shown.*

### Muscle-Induced COM Acceleration in the Natural Trials

**FIGURE 3:**
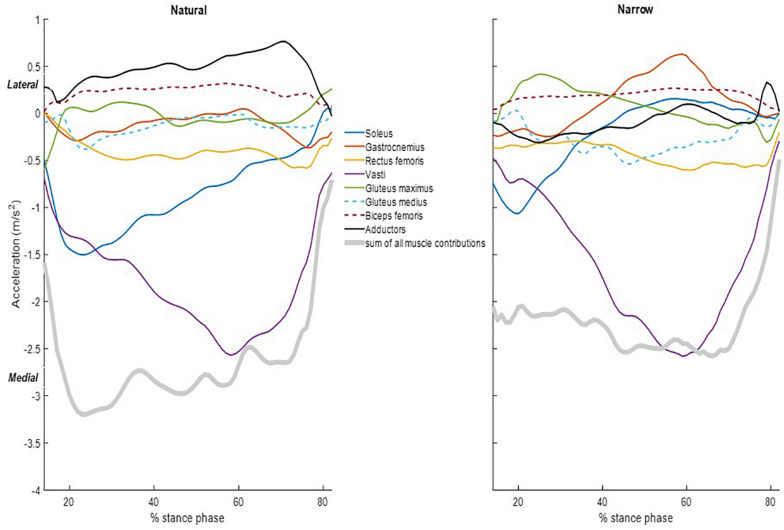
Individual muscle contributions and sum of all the muscle contributions to the medio-lateral COM acceleration during the stance phase in the natural (left) and narrow (right) trials. Data included are the average of all the subjects. Lower-limb muscles considered were soleus (blue), gastrocnemius (orange), rectus femoris (yellow), vasti (purple), gluteus maximus (green), gluteus medius (dashed light blue), biceps femoris long head (dashed maroon), and adductors (black). Muscle contributions are shown only between 14 and 82% of the stance phase.

The summed acceleration from all muscle contributions agreed with the measured acceleration, i.e., the summed muscle induced accelerations agreed with each sprinter’s GRF divided by body mass (Figure 2B). Individual muscle contributions were only reported between 14 and 82% of the stance phase due to computational limitations of the forward dynamics pipeline; the computed muscle control and induced acceleration steps cannot converge during the initial nor the final frames. Here, we refer to forward COM acceleration as “propulsion” and to vertical COM acceleration as “support,” as per (Hamner et al., 2010). The three vasti muscles are grouped together (vastus intermedius, lateralis and medialis), as are the adductors (adductor brevis, longus and magnus, semitendinosus, semimembranosus, and gracilis combined). The main contributor to propulsion was the soleus, followed by the gastrocnemius. The three vasti and the rectus femoris induced a rearward COM acceleration, i.e., decelerating forward progression. The main contributor to support was the soleus, followed by the summed contribution from the three vasti. Gastrocnemius, rectus femoris, gluteus maximus, and gluteus medius also contributed to support. Lastly, biceps femoris and adductors induced a downward COM acceleration, i.e., lowering the COM. The main contributor to medial COM acceleration was the vasti group, assisted by the soleus at the beginning of the stance phase (Figure 3). On the contrary, the adductors and the biceps femoris contributed to lateral COM acceleration.

**FIGURE 4:**
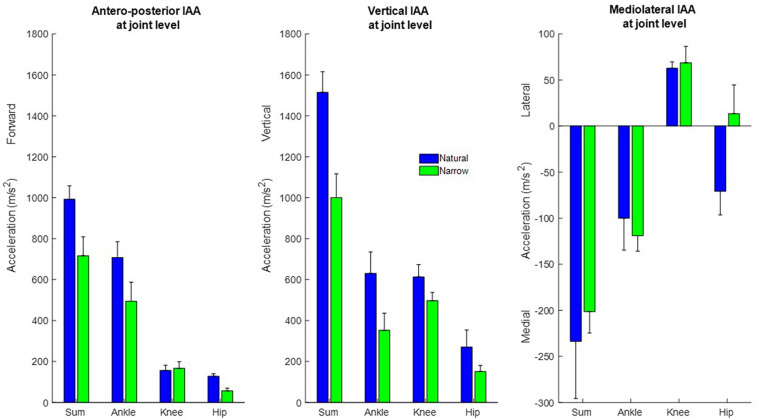
Cumulative contribution of all muscles, and of muscles spanning the ankle, knee, and hip, to the forward, vertical, and medio-lateral COM accelerations, in the natural (blue) and narrow (green) trials (mean ± SD). The cumulative induced accelerations at the joint level were computed as the sum of the cumulative accelerations during the stance phase induced by all muscles spanning that joint.

At the joint level, muscles around the ankle joint contributed most to propulsion and support followed by muscles around the knee and hip (Figure 4). The cumulative induced accelerations in the medial and lateral directions were much smaller than in other two directions. Muscles spanning the knee accelerated the COM medially, while muscles spanning the hip and ankle accelerated the COM laterally.

### Influence of Step Width in Muscle-Induced COM Acceleration

Compared to the natural trials, all muscles in general contributed less to propulsion in the narrow trials. The soleus had a distinctly smaller contribution to propulsion starting from approximately 25% of stance phase and the decreasing throughout the rest of stance. The gastrocnemius likewise contributed distinctly less to propulsion from approximately 59% of stance phase (Table 1). The vasti and rectus femoris also contributed less to rearward COM acceleration (i.e., decelerating forward progression) in the narrow trials. The vasti muscle group, rather than the soleus, was the main contributor to support in the narrow trials. The remaining muscles contributed less to support in the narrow trials than in the natural trials. The vasti group was again the main contributor to medial COM acceleration, to a similar amount as in the natural trials. The biceps femoris contributed again to lateral COM acceleration but the adductors contributed to lateral instead of medial COM acceleration in the narrow trials. The gluteus maximus contributed to lateral COM acceleration in early stance and the gastrocnemius, in late stance.

At the joint level, muscles spanning all joints induced less support and propulsion in the narrow trials with only one exception, that muscles spanning the knee induced slightly propulsion. Muscles spanning the knee contributed more to medial COM acceleration, and muscles spanning the ankle contributed to more lateral COM acceleration. Muscles spanning the hip induced a small medial COM acceleration in the narrow trials, rather than a lateral COM acceleration as in the natural trials.

## Discussion

The objectives of this study were to study individual muscle contributions to COM acceleration during a sprint start and to analyze whether the step width at the onset of sprint influenced the muscles’ induced accelerations. Muscle induced COM accelerations were computed from trials with the widest and the narrowest step width in four competitive sprinters. The results indicate that ankle plantarflexors, i.e., the soleus and the gastrocnemius, were the principal contributors to forward (propulsion) and vertical (support) COM acceleration, and that the vasti group was the dominant contributor to medial COM acceleration when athletes performed their natural wide sprint start. At the joint level, muscles contributed overall more to support than to propulsion in the first step of sprinting. When the step width was restricted, all muscles generally diminished their contributions to support and propulsion COM acceleration, most notably the ankle plantarflexors. The differences at the joint level were mostly evident in decreased contribution to support. The natural wide steps apparently provided a preferable body configuration to for the muscles to most effectively propel and support the COM in the sprint starts.

Our finding of dominance of the ankle plantarflexors in propulsion, not hip extensors intuitively, during the first stance of sprint running is in agreement with other studies (Debaere et al., 2013, 2015). The contraction of this muscle group in the first step when the COM is located anterior to the base of support provides an advantage that propels the sprinter forward. However, we found that the knee extensors, i.e., the vasti and the rectus femoris, decelerated forward COM progression, which contradicts findings of Debaere et al. (2015), who found through perturbation analysis that knee extensors contributed a small amount to forward COM acceleration. They also reported that knee joint moment contributed to COM propulsion only at the end of the first stance phase, which may explain the discrepancy to our findings, as we only computed induced accelerations until approximately 82% of stance phase. Our findings of knee extensor induced accelerations are in line with Hamner and Delp (2013), who reported that knee extensors decelerate the forward COM progression in early stance during running. Regarding support, the soleus was again the dominant contributor in the natural trials, followed closely by the vasti, similar to findings by Debaere et al. (2015). It is worthy noting that the knee joint is in a flexed position during the stance phase (highly flexed at touch down and reaching 20–30° at foot-off, Supplementary Material C), which makes the gastrocnemius less likely to contribute to ankle plantarflexion moment than the soleus. The major contribution of the knee extensors to support corroborates with findings from Jacobs and van Ingen Schenau (1992), and Charalambous et al. (2012); Debaere et al. (2013). In the mediolateral direction, the vasti group was the major contributor to medial COM acceleration, along with the soleus in early stance. These muscles largely control mediolateral balance, together with the adductors and the biceps femoris.

When the step width was reduced, all muscles’ contributions to COM propulsion and support were lower. Ankle plantarflexors and vasti were still the main contributors to anterior and medial COM acceleration, respectively. However, the vasti became the major contributors to support. This implies that in the narrower step position, the COM was lifted more through knee extension than through ankle plantarflexion. The spatio-temporal characteristics observed (Supplementary Material A) agree with muscle induced COM accelerations estimated by IAA. The magnitude of vertical and medial-lateral COM velocity was lower at toe-off in the narrow trials, but forward COM velocity was only marginally affected by the step width.

At the joint level, muscles spanning the ankle contributed somewhat more to propulsion than to support, whereas muscles spanning knee and ankle joint contributed more to support than propulsion. This observation agrees with findings from Debaere et al. (2013). It was reported that the first stance of sprint imposes demand that require technical skills in well-trained athletes favoring the knee joint rather than the ankle during the first stance phase of sprint (Charalambous et al., 2012; Debaere et al., 2013) and that the specific role of muscles that span the knee is to accelerate the COM vertically. Thus, lifting the COM is crucial for the first step of sprint. When the step width was restricted, muscles decreased their contribution to the COM accelerations. The greatest reduction was observed in support, wherein muscles spanning all joints induced less acceleration. The decreased contribution to propulsion was mainly due to the decreased acceleration induced by muscles spanning the ankle. Smaller contributions to forward and vertical COM acceleration imply that the restricted steps are not preferable for propelling and lifting the COM in sprint start. We observed that three of four participants had smaller ankle plantarflexion moment and a more flexed knee angle in the narrow trials (Supplementary Materials C,D). The combination of larger knee flexion with smaller ankle plantarflexion moment likely leads to the much smaller acceleration induced by the soleus during the narrow vs. natural trials. Very few studies have investigated sprint start in the transverse plan. One study claimed that minimizing the medial-lateral velocity of the pelvis seems to be optimal for development of forward velocity of COM (Debaere et al., 2013). In contrast to this argument, our findings suggest that a narrow step might indeed reduce the medial-lateral COM velocity, but no particular benefit in forward propulsion could be found; much the opposite, in fact.

There are several limitations in this study. First, there are computational limits when executing the computed muscle control and the induced acceleration analysis tools. The accelerations induced could not be analyzed during the first 0.03 s and the final 0.04 s, i.e., the first 14% and the last 18% of the stance phase, due to the abrupt changes in muscle forces and external forces in these phases. Secondly, the musculoskeletal model in OpenSim has several limitations. For instance, the generic model was a male model and there were two female sprinters in the participants; the knee joint is modeled as a hinge joint, which may affect the results; and the metatarsophalangeal (MTP) joint motion had to be ignored even though Bezodis et al. (2012) found that its exclusion would cause an unnaturally high peak in ankle joint moments in sprint running. The MTP joint was locked because the model could not accurately represent the motion of the toes even though there were enough markers to model this motion. Moreover, the findings in the study were based on four competitive sprinters. A larger cohort of participants will be helpful to generalize the results. In addition, muscle induced COM accelerations were not investigated during the block start phase due to the limited GRF recording. Finally, care should be taken when generalizing the results of the different contributors to COM acceleration, especially in the medial direction in both trials, as most variation between athletes was seen in this direction.

## Conclusion

Ankle plantarflexors, in particular the soleus, were the main contributors to propulsion regardless of step width. Ankle plantarflexors and knee extensors were the primary contributors to support. The vasti contributed most to medial COM acceleration, regardless of step width. Hip extensors and hip adductors had relatively small contributions to COM acceleration in any direction, regardless of step width, although the gluteus medius was found to contribute more in the narrow than in the natural trials. At the joint level, muscles spanning the ankle contributed mostly to propulsion, while muscles spanning the ankle and knee joint were the dominant contributors to support. When step width was restricted, practically all muscles contributed less to propulsion and support, which implies that narrow steps might inhibit the muscles spanning the ankle and particularly the knee to maximize their performance in sprint start. Based on our simulation, no muscular advantage was found with narrower step width.

## Data Availability Statement

The raw data supporting the conclusions of this article will be made available by the authors, without undue reservation.

## Ethics Statement

The studies involving human participants were reviewed and approved by the Stockholm Regional Ethical Committee. The patients/participants provided their written informed consent to participate in this study.

## Author Contributions

RW participated in the study design, interpretation of results and drafted the manuscript. LM performed the simulation and drafted the manuscript. PS initiated the project, and collected the motion data and participated in the interpretation of results. AA initiated the project and participated in the study design and interpretation of results. EG-F participated in the study design and interpretation of results. All authors contributed to the manuscript writing. All authors have read and approved the final version of the manuscript.

## Conflict of Interest

The authors declare that the research was conducted in the absence of any commercial or financial relationships that could be construed as a potential conflict of interest.
