# Supplementary material for: The Effect of Step Width on Muscle Contributions to Body Mass Center Acceleration During the First Stance of Sprinting
Source: Front Bioeng Biotechnol. 2021 Jul 14;9:636960. doi: 10.3389/fbioe.2021.636960 (PMC8318133; doi:10.3389/fbioe.2021.636960)
Supplement: Supplementary file 1 [file Data_Sheet_1.docx]

**Supplementary A**

Table A1. Comparison of kinematic (step width, velocity at toe-off, and stance phase time) and kinetic (normalized average antero-posterior power and maximum forward, vertical and lateral data in the natural and narrow trials during the first stance phase for every subject. The mean and its corresponding standard deviation values (in brackets) for each trial are also shown. Forces are measured in body weight (BW).

|  |  | **Male 1** | |  | **Male 2** | |  | **Female 1** | |  | **Female 2** | |  | **Average** | |
| --- | --- | --- | --- | --- | --- | --- | --- | --- | --- | --- | --- | --- | --- | --- | --- |
|  |  | **Natural** | **Narrow** |  | **Natural** | **Narrow** |  | **Natural** | **Narrow** |  | **Natural** | **Narrow** |  | **Natural** | **Narrow** |
| **Step width (m)** |  | 0.42 | 0.11 |  | 0.34 | 0.15 |  | 0.33 | 0.16 |  | 0.38 | 0.2 |  | 0.37 (0.04) | 0.16 (0.04) |
| **Velocity at toe-off (m/s)** | **Forward** | 4.31 | 4.44 |  | 4.41 | 4.29 |  | 4.54 | 4.46 |  | 4.23 | 4.24 |  | 4.37 (0.14) | 4.36 (0.11) |
|  | **Vertical** | 0.55 | 0.39 |  | 0.47 | 0.54 |  | 0.40 | 0.39 |  | 0.49 | 0.48 |  | 0.48 (0.06) | 0.45 (0.07) |
|  | **Lateral** | -0.02 | 0.01 |  | -0.11 | 0.00 |  | -0.16 | 0.01 |  | -0.01 | -0.01 |  | -0.08 (0.07) | 0.00 (0.01) |
| **Velocity at toe-on (m/s)** | **Forward** | 2.99 | 3.14 |  | 3.16 | 2.98 |  | 3.15 | 3.18 |  | 3.11 | 4.23 |  | 3.10 (0.08) | 3.38 (0.57) |
|  | **Vertical** | -0.06 | 0.06 |  | -0.08 | -0.14 |  | -0.15 | -0.16 |  | -0.19 | 0.49 |  | -0.12 (0.06) | 0.06 (0.30) |
|  | **Lateral** | 0.23 | -0.04 |  | 0.28 | 0.04 |  | 0.30 | 0.09 |  | 0.31 | -0.01 |  | 0.28 (0.04) | 0.02 (0.06) |
| **Change of velocity (m/s)** | **Forward** | 1.32 | 1.30 |  | 1.25 | 1.31 |  | 1.39 | 1.28 |  | 1.22 | 1.08 |  | 1.30 (0.08) | 1.24 (0.11) |
|  | **Vertical** | 0.61 | 0.33 |  | 0.55 | 0.68 |  | 0.55 | 0.55 |  | 0.68 | 0.49 |  | 0.60 (0.06) | 0.51 (0.15) |
|  | **Lateral** | -0.25 | 0.05 |  | -0.39 | -0.04 |  | -0.46 | -0.08 |  | -0.32 | -0.15 |  | -0.36 (0.09) | -0.06 (0.08) |
| **Stance phase time (s)** | | 0.19 | 0.23 |  | 0.21 | 0.19 |  | 0.21 | 0.24 |  | 0.19 | 0.22 |  | 0.20 (0.01) | 0.22 (0.02) |
| **Normalized average antero-posterior power^*^ (-)** | | 0.82 | 0.71 |  | 0.76 | 0.83 |  | 0.86 | 0.72 |  | 0.75 | 0.64 |  | 0.80 (0.05) | 0.73 (0.08) |
| **Max. forward force (BW)** | | 1.14 | 0.77 |  | 1.13 | 1.09 |  | 1.09 | 0.77 |  | 1.02 | 0.91 |  | 1.09 (0.05) | 0.89 (0.15) |
| **Max. rearward force (BW)** | | -0.02 | -0.4 |  | -1.06 | -0.57 |  | -0.4 | -0.59 |  | -0.52 | -0.71 |  | -0.50 (0.43) | -0.57 (0.13) |
| **Max. vertical force (BW)** | | 2.18 | 1.71 |  | 1.98 | 1.99 |  | 1.92 | 1.81 |  | 2.06 | 1.82 |  | 2.03 (0.12) | 1.83 (0.12) |
| **Max. medial force (BW)** | | -0.4 | -0.11 |  | -0.43 | -0.15 |  | -0.35 | -0.23 |  | -0.07 | -0.08 |  | -0.31 (0.16) | -0.14 (0.07) |
| **Max. lateral force (BW)** | | 0.06 | 0.03 |  | 0.1 | 0.17 |  | 0.10 | 0.02 |  | 0.47 | 0.24 |  | 0.18 (0.19) | 0.12 (0.11) |

^*^The average antero-posterior power ($\bar{P}_{AP}$) and normalised average antero-posterior power ($\bar{P}_{NAP}$) were computed during the first stance phase using the equations (1) and (2) (Bezodis et al., 2010; Sandamas et al., 2019):

| $\bar{P}_{AP}=\frac{m\cdot({v_{TO}}^{2}-{v_{TD}}^{2})}{2\cdot\Delta t}$ | (1) |
| --- | --- |
| $\bar{P}_{NAP}=\frac{\bar{P}_{AP}}{m\cdot g^{3/2}\cdot l^{1/2}}$ | (2) |

where $m$ is the mass of the subject, $v_{TO}$is the COM velocity at toe-off, $v_{TD}$is the COM velocity at touchdown, $\Delta t$ is the duration of the contact (stance phase time), $g$ is the gravity constant, and $l$ is the leg length of the subject (vertical coordinate of the hip joint center, computed during the standing reference trial).

**Supplementary B**

A sample of normalized EMGs in the natural and narrow trials during the whole recording. The normalized EMG data between the solid dash lines were used to constraint the muscle force estimation in CMC.

**Supplementary C**

Kinematics of hip, knee and ankle joint over the stance phase in the narrow and natural trials.

**Female 1**

**Female 2**

**Male 1**

**Male 2**

**Supplementary D**

Kinematics of hip, knee and ankle joint over the stance phase in the narrow and natural trials.

**Female 1**

**Female 2**

**Male 1**

**Male 2**
